# Supplementary material for: Energy-Based Coarse-Graining in Molecular Dynamics: A Flow-Based Framework without Data
Source: J Chem Theory Comput. 2025 Oct 24;22(1):181–200. doi: 10.1021/acs.jctc.5c01504 (PMC12805520; doi:10.1021/acs.jctc.5c01504)
Supplement: Supplementary file 1 [file ct5c01504_si_001.pdf]

Supporting Information for Publication: Energy-Based Coarse-Graining in Molecular Dynamics: A Flow-Based Framework without Data

Maximilian Stupp† and P.S. Koutsourelakis*,†,‡

†*Professorship of Data-driven Materials Modeling, School of Engineering and Design, Technical University of Munich, Garching b. München 85748, Germany*

‡ *Munich Data Science Institute (MDSI - Core Member), Technical University of Munich, Garching b. München 85748, Germany*

E-mail: p.s.koutsourelakis@tum.de

Computation of the information-theoretic criterion used for the adaptive tempering scheme

The following note provides a derivation of the information-theoretic criterion used for the adaptive tempering scheme proposed in Section 2.2 of the main text and associated computational details.

Based on Equation (9) (main text), we can write the numerator and denominator in

Equation (22) (main text) that provide δKL_k as:

$$KL(q_{\boldsymbol{\theta}}(\mathbf{X},\mathbf{z})||p_{\boldsymbol{\phi}}(\mathbf{X},\mathbf{z};\ \beta_{k+1})) - KL(q_{\boldsymbol{\theta}}(\mathbf{X},\mathbf{z})||p_{\boldsymbol{\phi}}(\mathbf{X},\mathbf{z};\ \beta_k)) = \Delta\beta_k \left\langle U_{\boldsymbol{\phi}}(\mathbf{X},\mathbf{z}\ ;\beta)\right\rangle_{q_{\boldsymbol{\theta}}(\mathbf{X},\mathbf{z})} + \log\frac{Z_{\beta_{k+1}}}{Z_{\beta_k}} \quad (1)$$

and

$$KL(q_{\boldsymbol{\theta}}(\mathbf{X},\mathbf{z})||p_{\boldsymbol{\phi}}(\mathbf{X},\mathbf{z}\ ;\beta_k)) = \beta_k \left\langle U_{\boldsymbol{\phi}}(\mathbf{X},\mathbf{z}\ ;\beta)\right\rangle_{q_{\boldsymbol{\theta}}(\mathbf{X},\mathbf{z})} + \log Z_{\beta_k} + \left\langle \log q_{\boldsymbol{\theta}}(\mathbf{X}|\mathbf{z})\right\rangle_{q_{\boldsymbol{\theta}}(\mathbf{X},\mathbf{z})} + \left\langle \log\ q_{\boldsymbol{\theta}}(\mathbf{z})\right\rangle_{q_{\boldsymbol{\theta}}(\mathbf{z})} \quad (2)$$

The computation of the terms excluding the partition functions, as discussed in the main text, can be carried out with Monte Carlo or analytically due to the form of $q_{\boldsymbol{\theta}}$ detailed. For the partition function Z_{β_k} and given the proximity of the optimized $q_{\boldsymbol{\theta}}(\mathbf{X},\mathbf{z})$ to $p_{\boldsymbol{\phi}}(\mathbf{X},\mathbf{z}\ ;\beta_k)$ we employ Importance Sampling (IS) with $q_{\boldsymbol{\theta}}$ as the IS density.[1] In particular:

$$\begin{aligned}
\log Z_{\beta_k} &= \log \int e^{-\beta_k U_{\boldsymbol{\phi}}(\mathbf{X},\mathbf{z}\ ;\beta)}\ d\mathbf{X}\ d\mathbf{z} \\
&= \log \int \frac{e^{-\beta_k U_{\boldsymbol{\phi}}(\mathbf{X},\mathbf{z}\ ;\beta_k)}}{q_{\boldsymbol{\theta}}(\mathbf{X},\mathbf{z})} q_{\boldsymbol{\theta}}(\mathbf{X},\mathbf{z})\ d\mathbf{X}\ d\mathbf{z} \\
&\approx \log\left(\frac{1}{N}\sum_{i=1}^{N} w^{(i)}\right)
\end{aligned} \quad (3)$$

where $w^{(i)}$ denote the *unnormalized* IS weights computed as:

$$w^{(i)} = \frac{e^{-\beta_k U_{\boldsymbol{\phi}}(\mathbf{X}^{(i)},\mathbf{z}^{(i)};\ \beta_k)}}{q_{\boldsymbol{\theta}}(\mathbf{X}^{(i)},\mathbf{z}^{(i)})} \quad (4)$$

where $(\mathbf{X}^{(i)},\mathbf{z}^{(i)})$ are i.i.d samples drawn from $q_{\boldsymbol{\theta}}$. To avoid numerical underflow, we operate with the log-weights shifted by their maximum, i.e. $\tilde{w}^{(i)} = \log w^{(i)} - \log w_{max}$ where

$\log w_{\max} = \max_i \log w^{(i)}$ and use the following estimator:

$$\log Z_{\beta_k} \approx \log \left(\frac{e^{\log w_{max}}}{N} \sum_{i=1}^{N} e^{\tilde{w}^{(i)}} \right) = \log w_{max} - \log N + \log \left(\sum_{i}^{N} e^{\tilde{w}^{(i)}} \right) \tag{5}$$

Finally, with regard to the $\log \frac{Z_{\beta_{k+1}}}{Z_{\beta_k}}$ term, we have:

$$\begin{aligned}
\log Z_{\beta_{k+1}} &= \log \int e^{-\beta_{k+1} U_{\boldsymbol{\phi}}(\mathbf{X},\mathbf{z}\ ;\beta_{k+1})}\ d\mathbf{X}\ d\mathbf{z} \\
&= \log \int \frac{e^{-(\beta_k+\Delta\beta_k) U_{\boldsymbol{\phi}}(\mathbf{X},\mathbf{z};\ \beta_{k+1})}}{\frac{e^{-\beta_k U_{\boldsymbol{\phi}}(\mathbf{X},\mathbf{z};\ \beta_k)}}{Z_{\beta_k}}} \frac{e^{-\beta_k U_{\boldsymbol{\phi}}(\mathbf{X},\mathbf{z}\ ;\beta_k)}}{Z_{\beta_k}}\ d\mathbf{X}\ d\mathbf{z} \\
&= \log Z_{\beta_k} + \log \int e^{-\Delta\beta_k U(\boldsymbol{f}_{\boldsymbol{\phi}}(\mathbf{X},\mathbf{z}))} \frac{e^{-\beta_k U_{\boldsymbol{\phi}}(\mathbf{X},\mathbf{z}\ ;\beta_k)}}{q_{\boldsymbol{\theta}}(\mathbf{X},\mathbf{z})} \frac{q_{\boldsymbol{\theta}}(\mathbf{X},\mathbf{z})}{Z_{\beta_k}}\ d\mathbf{X}\ d\mathbf{z}. \\
&\qquad \text{(from Equation (5) in the manuscript)}
\end{aligned} \tag{6}$$

Hence:

$$\begin{aligned}
\log \frac{Z_{\beta_{k+1}}}{Z_{\beta_k}} &= \log \int e^{-\Delta\beta_k U(\boldsymbol{f}_{\boldsymbol{\phi}}(\mathbf{X},\mathbf{z}))} w(\mathbf{X},\mathbf{z}) \frac{q_{\boldsymbol{\theta}}(\mathbf{X},\mathbf{z})}{Z_{\beta_k}}\ d\mathbf{X}\ d\mathbf{z} \\
&\approx \log \left(\frac{1}{N} \sum_{i=1}^{N} e^{-\Delta\beta_k U(\boldsymbol{f}_{\boldsymbol{\phi}}(\mathbf{X}^{(i)},\mathbf{z}^{(i)}))} \frac{w^{(i)}}{Z_{\beta_k}} \right) \\
&= \log \left(\sum_{i=1}^{N} e^{-\Delta\beta_k U(\boldsymbol{f}_{\boldsymbol{\phi}}(\mathbf{X}^{(i)},\mathbf{z}^{(i)}))} W^{(i)} \right)
\end{aligned} \tag{7}$$

where $W^{(i)} = \frac{w^{(i)}}{\sum_{j=1}^{N} w^{(j)}}$ are the *normalized* IS weights. As before, we can employ the shift of the log-weights to avoid numerical underflow problems in the computation of $W^{(i)}$.

References

(1) Liu, J. *Monte Carlo Strategies in Scientific Computing*; Springer Series in Statistics; Springer.
